# Hypercapnia attenuates ventilator-induced diaphragm atrophy and modulates dysfunction

**DOI:** 10.1186/cc13719

**Published:** 2014-02-09

**Authors:** Willem-Jan M Schellekens, Hieronymus WH van Hees, Matthijs Kox, Marianne Linkels, Gilberto L Andrade Acuña, PN Richard Dekhuijzen, Gert Jan Scheffer, Johannes G van der Hoeven, Leo MA Heunks

**Affiliations:** 1 Department of Anesthesiology, Radboud University Medical Centre, Geert Grooteplein Noord 9, 6500 HB Nijmegen, The Netherlands; 2 Pulmonary Diseases, Radboud University Medical Centre, Geert Grooteplein Noord 9, 6500 HB Nijmegen, The Netherlands; 3 Intensive Care Medicine, Radboud University Medical Centre, Geert Grooteplein Noord 9, 6500 HB Nijmegen, The Netherlands

## Abstract

**Introduction:**

Diaphragm weakness induced by prolonged mechanical ventilation may contribute to difficult weaning from the ventilator. Hypercapnia is an accepted side effect of low tidal volume mechanical ventilation, but the effects of hypercapnia on respiratory muscle function are largely unknown. The present study investigated the effect of hypercapnia on ventilator-induced diaphragm inflammation, atrophy and function.

**Methods:**

Male Wistar rats (n = 10 per group) were unventilated (CON), mechanically ventilated for 18 hours without (MV) or with hypercapnia (MV + H, Fico_2_ = 0.05). Diaphragm muscle was excised for structural, biochemical and functional analyses.

**Results:**

Myosin concentration in the diaphragm was decreased in MV versus CON, but not in MV + H versus CON. MV reduced diaphragm force by approximately 22% compared with CON. The force-generating capacity of diaphragm fibers from MV + H rats was approximately 14% lower compared with CON. Inflammatory cytokines were elevated in the diaphragm of MV rats, but not in the MV + H group. Diaphragm proteasome activity did not significantly differ between MV and CON. However, proteasome activity in the diaphragm of MV + H was significantly lower compared with CON. LC3B-II a marker of lysosomal autophagy was increased in both MV and MV + H. Incubation of MV + H diaphragm muscle fibers with the antioxidant dithiothreitol restored force generation of diaphragm fibers.

**Conclusions:**

Hypercapnia partly protects the diaphragm against adverse effects of mechanical ventilation.

## Introduction

It is well known that controlled mechanical ventilation adversely affects the respiratory muscles [1,2]. For instance, Levine and colleagues demonstrated that, in brain-dead patients, 18 to 69 hours of mechanical ventilation is associated with profound atrophy of the diaphragm [1]. Enhanced proteolysis [3] and proinflammatory cytokines [4] have been implicated in the development of ventilator-induced diaphragm dysfunction.

Hypercapnia is a well-tolerated side effect of low tidal volume ventilation in patients with acute respiratory distress syndrome. Moreover, experimental studies indicate that hypercapnic acidosis may exert beneficial effects by reducing inflammation and lung injury during mechanical ventilation [5-8]. For instance, in rats with ventilator-induced lung injury, hypercapnic acidosis reduced interleukin (IL)-6 and tumor necrosis factor alpha (TNF-α) in bronchoalveolar lavage fluid compared with normocapnia [6]. Recently, Jung and colleagues demonstrated that in pigs hypercapnic acidosis prevents the loss of diaphragm force induced by controlled mechanical ventilation [9]. The latter study was largely descriptive and the effects of hypercapnic acidosis on downstream mechanisms of ventilator-induced diaphragm dysfunction remain largely unknown [10]. Inflammatory and oxidative pathways have been implicated in the development of skeletal muscle atrophy and dysfunction [4,9-12]. For instance, we have reported that IL-6 may be an important mediator in sepsis-induced skeletal muscle atrophy [11].

Accordingly, the following hypotheses were tested in the present study: first, hypercapnic acidosis attenuates ventilator-induced diaphragm atrophy and dysfunction. Second, hypercapnic acidosis inhibits upregulation of inflammatory cytokines and proteolysis in the diaphragm of mechanically ventilated rats.

To test these hypotheses healthy rats were randomized to an unventilated group, controlled mechanical ventilation during normocapnia or mechanical ventilation under hypercapnic conditions. Diaphragm atrophy, function, inflammation and proteolysis were assessed. Based on these results, additional experiments were performed to assess the role of oxidative protein modification on contractile function of the diaphragm.

## Materials and methods

### Design

Animal experiments were approved by the Regional Animal Ethics Committee (Nijmegen, The Netherlands) and performed under the guidelines of the Dutch Council for Animal Care.

Experiments were performed in male Wistar rats (Harlan, Horst, The Netherlands) randomly divided in three groups (n = 10 per group): control group (CON, bodyweight 313 ± 20 gram), mechanical ventilation group (MV, bodyweight 294 ± 15 gram) and mechanical ventilation with hypercapnia group (MV + H, bodyweight 313 ± 12 gram). The CON group was anesthetized with pentobarbital (50 mg/kg) and sacrificed without being mechanically ventilated.

Rats allocated to mechanical ventilation were anesthetized with an intraperitoneal injection of pentobarbital (induction 50 mg/kg), orally intubated and ventilated (ventilator UB 7025 from Hugo Sachs, March-Hugstetten, Germany) as described previously [13]. Briefly, in all mechanically ventilated animals, a catheter was inserted under sterile conditions in the carotid artery for continuous measurement of blood pressure and periodic blood sampling (at ½, 2, 4, 8, 12, 16 and 18 hours; i-STAT, Blood Gas Analyzer, Abbot, Hoofddorp, The Netherlands). Tidal volume was set at 6 ml/kg bodyweight, respiratory rate of 110/min, positive end-expiratory pressure of 1.5 cmH_2_O and inspired oxygen fraction of 0.45. Hypercapnic acidosis was induced by adding Fico_2_ 0.05 in the MV + H group. Ventilated rats were fed isocaloric AIN-76 rodent diet (SSNIFF Spezialdiäten, Soest, Germany) through an orogastric tube and received a continuous intravenous dose of pentobarbital (10 mg/kg/hour) via a tail vein catheter as previously described [14]. To compensate for loss of circulating volume, Ringer’s solution at 2 ml/hour was administered intravenously. Body temperature was kept between 36.0°C and 37.0°C using a heating pad [13]. All animals survived the 18 hours of mechanical ventilation without complications.

### Tissue collection

Immediately after anesthesia (CON) or after 18 hours of mechanical ventilation (MV and MV + H groups), rats were exsanguinated and a combined thoracotomy and laparotomy was performed, as described previously [13]. Left and right hemidiaphragm tissue was rinsed with the left part quickly frozen in liquid nitrogen and stored at -80°C for later biochemical analysis and the right hemidiaphragm submersed in cooled Krebs solution at pH 7.4 for single fiber isolation, as described previously [15].

### Myosin heavy chain isoform and concentration

Content of myosin heavy chain (MyHC) was analyzed in diaphragm muscle homogenates using standard Western blotting as described previously [4,16]. MyHC content on blot was corrected for loaded amount of muscle weight. For a more accurate determination of MyHC content in diaphragm, MyHC concentration (that is amount of myosin per muscle volume) was measured in diaphragm single fibers. Determination of MyHC isoform composition and concentration in the same diaphragm fibers as used for contractile measurements by means of SDS-PAGE was described previously [17] and adapted from Geiger *et al*. [18]. Briefly, single fibers were placed in a sample buffer, thereafter run on gel and afterward silver stained and quantified. Since only five diaphragm fibers in each group expressed the slow isoform of MyHC, these were excluded from further analysis. Accordingly, all fibers were classified as fast (2x and 2b) type fibers.

### Skinned fiber: cross-sectional area, contractile measurements

Cross-sectional area (CSA) and maximal active force generation of skinned single fibers isolated from the diaphragm muscle were determined (five to six fibers per rat) as described previously [17].

To obtain single fibers, a rectangular bundle from the central costal region of the right hemidiaphragm was dissected, parallel to the long axis of the muscle fibers. The muscle bundle was chemically skinned, that is permeabilization of lipid membranes. Subsequently, single fibers were isolated from the muscle bundle, attached to aluminum foil clips, and mounted in a flow-through acrylic chamber on two hooks connected to a force transducer (model AE-801; SensoNor, Horten, Norway) and a servomotor (model 308B, Aurora Scientific, Aurora, ON, Canada). Sarcomere length was set at 2.4 μm as the optimal length for force generation. Muscle fiber CSA area was deduced from fiber width and depth measurements using a reticule in the microscope eyepiece. Maximum isometric force was determined by measuring force after perfusing the experimental chamber with, successively, pCa 9.0 and pCa 4.5 solutions. Maximum specific force was derived from dividing maximum isometric force by fiber CSA [19].

### Cytokines

Concentrations of IL-1beta, IL-6, keratinocyte-derived chemokine (KC), IL-10 and TNF-α in diaphragm homogenates were analyzed with ELISA as described previously (R&D Systems, Minneapolis, MN, USA) [4].

### E3-ligases, ubiquitin-proteasome pathway, autophagy and transcription

To assess involvement of proteolysis we measured 20S proteasome proteolytic activity as described previously [16]. Levels of muscle-specific E3-ligases, markers for muscle atrophy, MAFbx and MuRF-1 mRNA were determined using qPCR as previously described [16]. Concentration of ubiquitinated myosin and total myosin were determined as described before in whole diaphragm muscle homogenates, using standard Western blotting [4,16]. To study the role of lysosomal autophagy, the content of autophagy markers light chain 3B-II (LC3B-II) and beclin-1 were measured by standard Western blotting as described previously [4], using specific antibodies against LC3B (anti-LC3B-II 2775, Cell Signaling Technology, Danvers, MA, USA) and beclin-1 (anti-beclin-1, PRS3613, Sigma-Aldrich, St Louis, MO, USA). Gels were equally loaded with 40 μg protein per sample. Optical density (OD) of LC3B-II and beclin-1 bands on blot were quantified using Odyssey scan and Odyssey application software version 2.1 (LI-COR Biosciences, Lincoln, NE, USA). In addition, MyHC expression of isoforms I, IIa, IIb and IIx was analyzed according to methods described for MAFbx and MuRF-1 mRNA. Forward and reverse oligonucleotides used were as follows: MyHC I: 5′–GCCAAGAGCCGTGACATTGGC–3′ and 5′–CTGCCTGAAGGTGCTGTTTCA –3′, MyHC IIa 5′–TATCCTCAGGCTTCAAGATTTG–3′ and 5′–TAAATAGAATCACATGGGGACA–3′, MyHC IIB 5′CACACCAAAGTCATAAG CGAA–3′ and 5′–CCTTGATATACAGGACAGTGA–3′, MyHC IIX 5′–TGATCGATCCAAAGCAGG–3′ and 5′–CTCCCAAAGTCGTAAGTA–3′.

### Effect of oxidation/reduction state on force generation and the presence of oxidative stress

To investigate involvement of reversible protein modifications by oxidative agents, additional experiments were performed using reducing agent dithiothreitol (DTT) (Sigma-Aldrich, Zwijndrecht, The Netherlands). Maximal active force generation of skinned diaphragm fibers (n = 16) was determined before and after 20 min incubation with DTT (10 mM in relaxing solution) [20].

To investigate the presence of oxidative stress, HNE (4-hydroxy-2-nonenal) was measured using a specific antibody against HNE (anti-HNE 393206, Calbiochem, Darmstadt, Germany). Diaphragm muscle homogenates were prepared as described previously [4]. Optical density of HNE bands on blot were quantified using Odyssey scan and Odyssey application software version 2.1 (LI-COR Biosciences, Lincoln, NE, USA).

### Data treatment and statistical methods

Differences among groups were analyzed with one-way ANOVA and Student-Newman-Keuls *post hoc* testing if appropriate. A two-sided paired Student’s *t* test was performed to evaluate the statistical difference between maximal forces before and after DTT incubation. Difference among groups regarding time courses of pH, Paco_2_, Pao_2_ and mean arterial pressure (MAP) were performed with a two-way ANOVA. GraphPad Prism was used to conduct statistical analysis (GraphPad Software Inc., San Diego, CA, USA). A probability level of *P* <0.05 was considered statistically significant. All data are presented as mean ± SE, except bodyweight that is presented as mean ± SD.

## Results

### Animal characteristics

**Figure 1:**
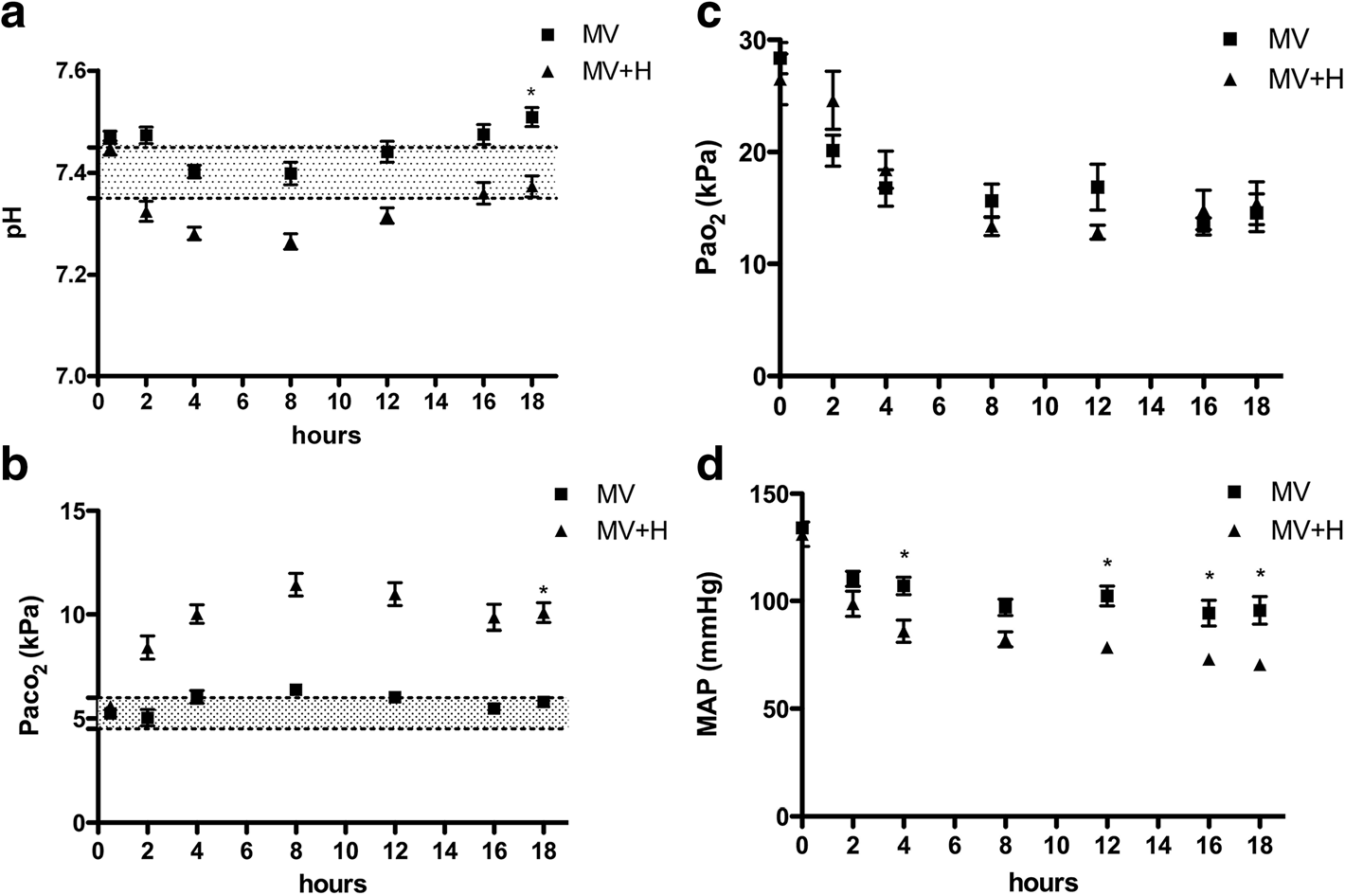
**Course of blood pH, CO**_**2,**_**O**_**2 **_**and MAP during 18 hours of mechanical ventilation. (a)** As expected increasing Fico_2_ reduces blood pH. *Two-way repeated measure analysis showed that pH values over time were significantly different between groups. **(b)** CO_2_ was significantly increased at the end of experiment in hypercapnic rats. *Two-way repeated measure analysis showed that CO_2_ values over time were significantly different between groups. **(c)** O_2_ was not significantly different between mechanically ventilated groups. **(d)** MAP was significantly lower at 4 hours and at the end of experiment in hypercapnic rats. *Two-way repeated measure analysis showed that MAP was significantly different between groups at 4, 12, 16 and 18 hours of mechanical ventilation. MAP, mean arterial pressure.

Animals allocated to ventilated groups were hemodynamically stable during the 18 hours of ventilation. No respiratory efforts were observed clinically or from the pressure tracing of the ventilator. Figure 1a-b-c-d shows the course of pH, Paco_2_, Pao_2_ and MAP during mechanical ventilation. As expected MV + H rats developed respiratory acidosis that was partly metabolically compensated. Plasma HCO_3_^-^ after 18 hours of mechanical ventilation was 33.6 ± 0.7 mmol/L in MV and 43.7 ± 3.1 mmol/L in MV + H.

### Hypercapnia and diaphragm structure and function

**Figure 2:**
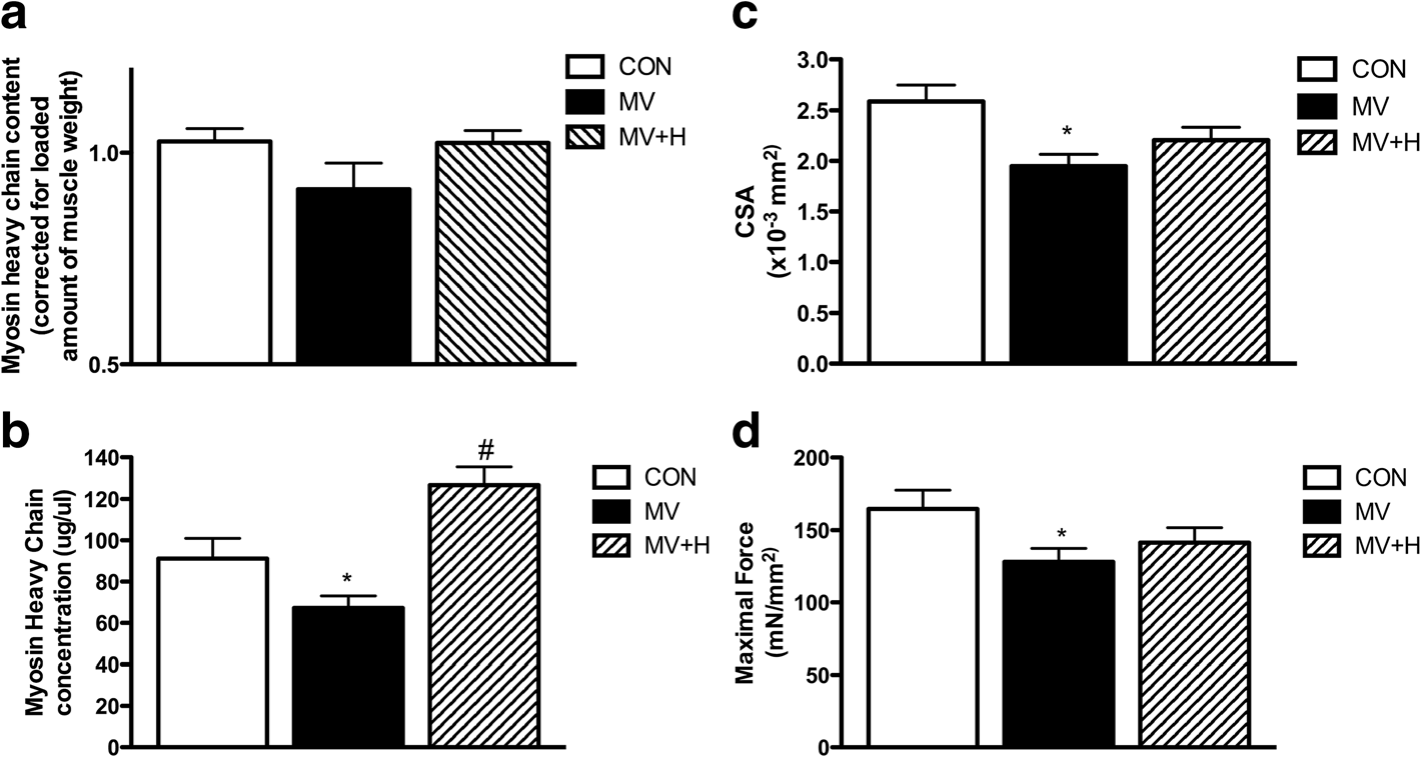
**Diaphragm myosin heavy chain content and concentration, cross-sectional area and function after 18 hours of mechanical ventilation. (a)** Myosin heavy chain content per muscle weight in diaphragm of the MV group showed a decreased trend compared with CON and MV + H, but was not significantly different between groups. **(b)** Myosin heavy chain concentration in diaphragm single fibers. Control diaphragm fibers (n = 51) showed a higher concentration of myosin heavy chain compared with fibers from ventilated rats (n = 57) (*versus control; *P* <0.05). Fibers from hypercapnic rats (n = 53) showed higher concentration of myosin heavy chain versus fibers from normocapnic ventilated animals (#versus MV and versus control; *P* <0.05). **(c)** Diaphragm fast type fibers cross-sectional area. Cross-sectional area was reduced in MV animals versus CON (*P <0.05). The reduction of cross-sectional area was less pronounced in diaphragm fibers from MV + H rats. **(d)** Maximal force corrected for cross-sectional area. Force of fibers from 18-hour mechanically ventilated rats were significantly lower compared with control (*P <0.05). CON, control; MV, mechanically ventilated; MV + H, mechanically ventilated with hypercapnia.

Hypercapnia prevented the loss of myosin in ventilated rats (Figure 2a). Subsequently, for a more precise measurement of muscle atrophy, MyHC concentration was measured in diaphragm single fibers. Accordingly, mechanical ventilation significantly decreased myosin concentration in diaphragm muscle fibers (Figure 2b). Hypercapnia prevented the loss of myosin induced by mechanical ventilation; in fact, mean myosin concentration was higher in diaphragm fibers from the MV + H group compared with the CON group (Figure 2b).

Besides myosin concentration, muscle fiber CSA was measured as an additional marker for atrophy. Diaphragm fiber CSA was reduced by approximately 25% in MV animals (Figure 2c, *P* <0.05 versus CON). The reduction of CSA was less pronounced, that is approximately 15%, in diaphragm fibers from MV + H rats (*P* = 0.06, versus CON).

Mechanical ventilation reduced specific force generation of diaphragm muscle fibers by approximately 22% compared with CON (Figure 2d, *P* <0.05). The force-generating capacity of diaphragm fibers from the MV + H group was approximately 14% lower compared with CON, but this difference did not reach statistical significance (*P* = 0.16).

### Inflammation

**Figure 3:**
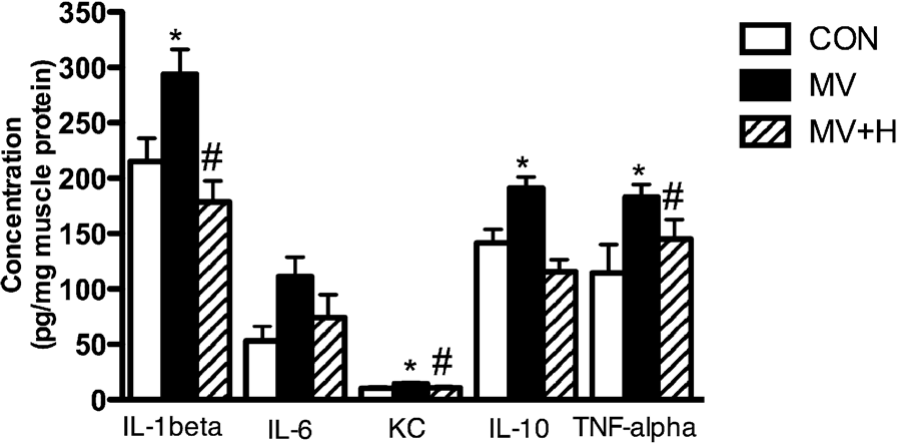
**Cytokine levels in diaphragm homogenates.** Mechanical ventilation significantly increased levels of interleukin (IL)-1beta, keratinocyte-derived chemokine (KC), IL-10 and tumor necrosis factor alpha (TNF-α) (*versus control; *P* <0.05). Hypercapnia largely abolished ventilator-induced inflammation in the diaphragm after 18 hours of MV (#versus MV; *P* <0.05 for IL-1beta, KC and TNF-α). MV + H versus CON was not significantly different for all cytokines. MV, mechanically ventilated; MV + H, mechanically ventilated with hypercapnia.

Mechanical ventilation resulted in increased levels of inflammatory cytokines in the diaphragm. For instance, IL-1beta, IL-6 and TNF-α increased by 37%, 109%, and 35%, respectively compared with CON. This inflammatory response was largely abolished in MV + H animals (Figure 3).

### E3-ligases, ubiquitin-proteasome pathway, autophagy and transcription

**Figure 4:**
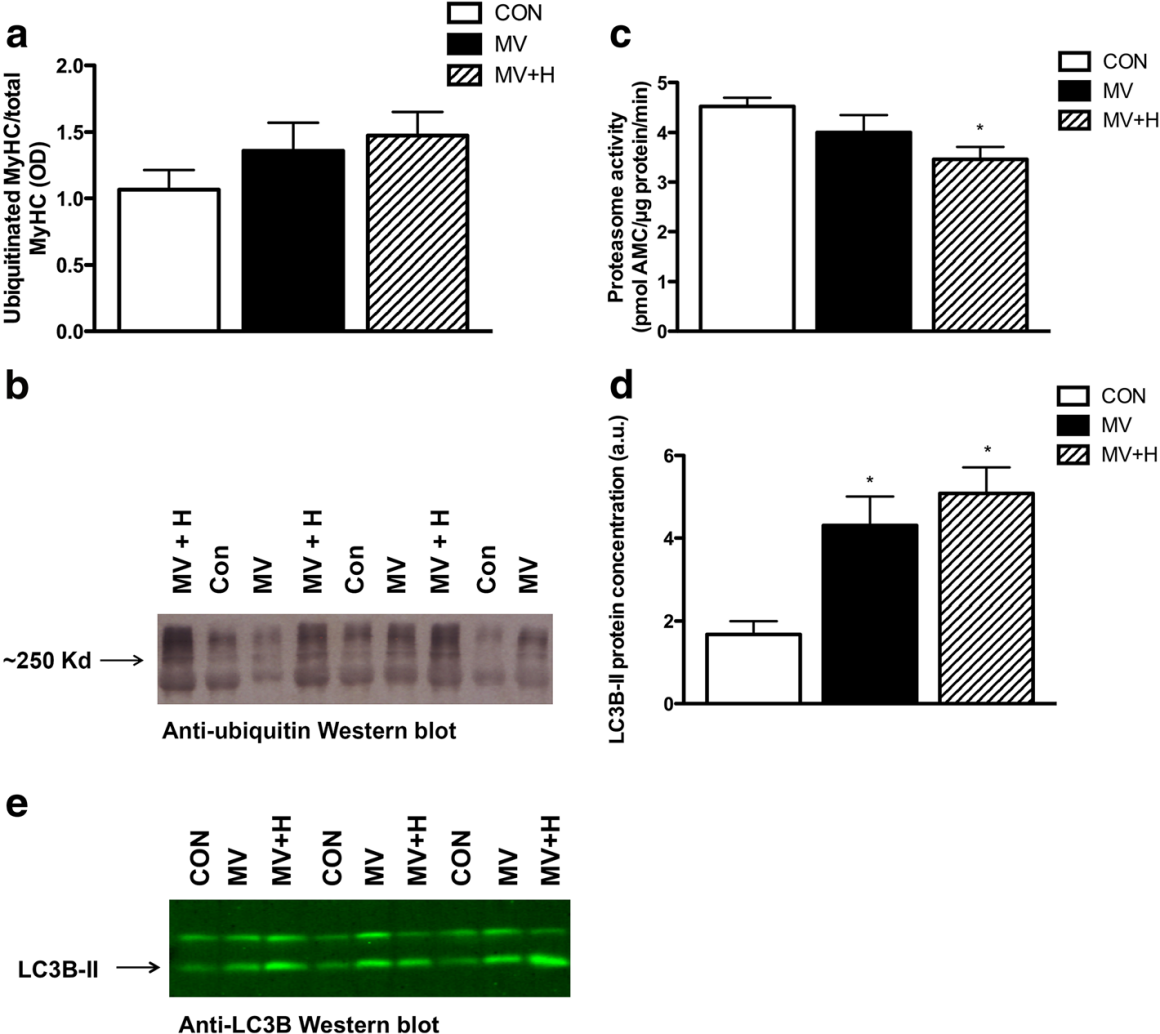
**Diaphragm ubiquitinated myosin and representative blot, proteasome activity, LC3B-II content and representative blot. (a)** Concentration of ubiquitinated myosin per total myosin in diaphragm of MV and MV + H groups showed an increased trend compared with control, but was not significantly different between groups. **(b)** Representative Western blot of ubiquitinated myosin. **(c)** 20S proteasome activity in diaphragm. Hypercapnic ventilated animals demonstrate decreased activity versus control animals (*P <0.05). **(d)** LC3B-II content. Both ventilated and hypercapnic ventilated animals were significantly different compared with control (*P <0.05). **(e)** Representative Western blot against LC3B-II. LC3B-II, light chain 3B-II; MV, mechanically ventilated; MV + H, mechanically ventilated with hypercapnia.

**Figure 5:**
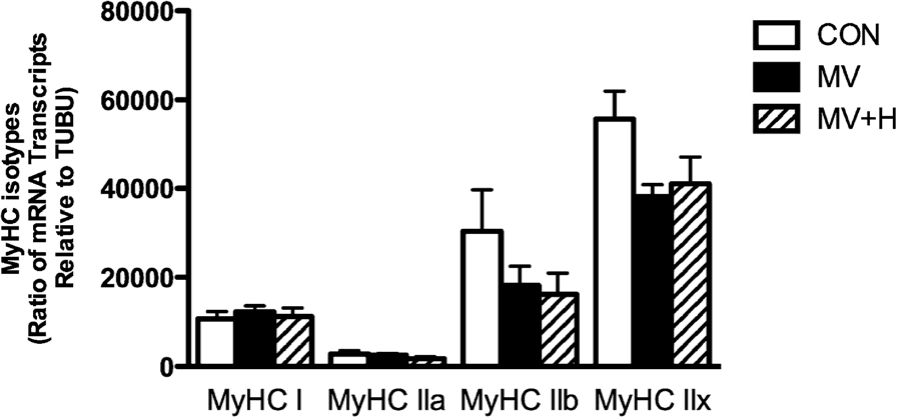
**Expression of mRNA myosin heavy chain isoforms I, IIa, IIb and IIx per tubulin in rat diaphragm.** Transcription was not different between groups.

To test whether the protective effects of hypercapnia on diaphragm myosin concentration in the ventilation group were mediated by inhibition of proteolytic pathways, we subsequently analyzed essential segments of the ubiquitin-proteasome pathway and a key lysosomal autophagy marker. Mechanical ventilation significantly enhanced mRNA expression of the E3-ligases MuRF-1 (11.4-fold expression in MV versus CON, *P* <0.05) and MAFbx (11.9-fold expression in MV versus CON, *P* <0.05) in the diaphragm. Hypercapnia did not affect ventilator-induced activation of these E3-ligases in the diaphragm (13.1-fold increase in MuRF-1 and 9.1-fold increase in MAFbx versus CON, both *P* <0.05). A trend for an increased amount of ubiquitinated myosin molecules was found in MV (Figure 4a/b, 128% in MV versus CON) and MV + H group (138% in MV + H versus CON) compared with control, but these differences did not reach statistical significance. Diaphragm proteasome activity was not significantly different between MV and CON (Figure 4c). However, proteasome activity in the diaphragm of the hypercapnic ventilated group was 24% lower compared with CON *P* <0.05 (Figure 4c). LC3B-II, a marker for autophagy, was increased in both ventilated groups compared with CON (Figure 4d/e). Another marker for autophagy, beclin-1, was not different between groups. Myosin expression in relation to the expression of tubulin was not different between groups for MyHC isoforms I, IIa, IIb and IIx (Figure 5).

### Effect of oxidation/reduction state on force generation

An unexpected finding was that the protective effect of hypercapnia on contractile protein concentration was only partly accompanied by a protective effect on force generation (see Figure 2b and 2d respectively).

**Figure 6:**
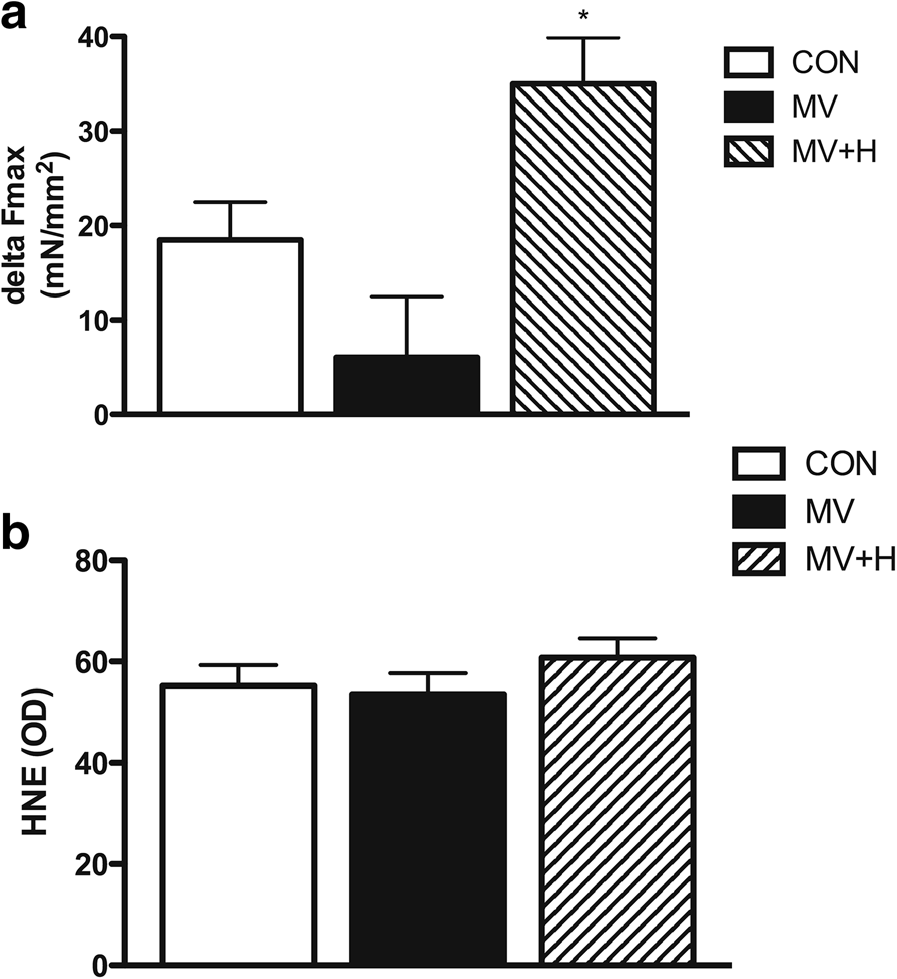
**Delta force, corrected for cross-sectional area, in single fibers before and after incubation with DTT and diaphragm 4-hydroxy-2-nonenal optical density. (a)** Delta force in MV + H (both n = 16) versus CON and MV groups was significant increased (*P <0.05). **(b)** 4-hydroxy-2-nonenal was not significantly different between groups. CON, control; DTT, dithiothreitol; MV, mechanically ventilated; MV + H, mechanically ventilated with hypercapnia.

Reduced force generation without the loss of contractile protein in hypercapnic rats suggests posttranslational modifications of the contractile proteins. Oxidative modifications are known to affect respiratory muscle force and indeed mechanical ventilation has been associated with oxidative stress [12,21]. Accordingly, we investigated if reversible protein oxidation is involved in the reduced force-generating capacity of diaphragm fibers in the MV + H group. Incubation of diaphragm muscle fibers from hypercapnic ventilated rats with the antioxidant DTT increased force generation by 27% (*P* <0.05 versus CON, Figure 6a). In contrast, DTT did not affect force generation of diaphragm fibers from the MV group (Figure 6a). Concentration of HNE, a marker for reversible oxidative stress, in diaphragm was not different between groups (Figure 6b).

## Discussion

Previous studies have shown that controlled mechanical ventilation has detrimental effects on diaphragm structure and function. In the present study, we report beneficial effects of hypercapnic acidosis on the diaphragm during controlled mechanical ventilation. The most important new findings are that 1) mechanical ventilation under hypercapnic conditions prevents loss of myosin in the diaphragm. 2) Hypercapnic acidosis largely abolishes ventilator-induced inflammation in the diaphragm. 3) Hypercapnic acidosis does not attenuate the immediate effects of mechanical ventilation on diaphragm single fiber function. 4) The antioxidant DTT restores diaphragm muscle function in the hypercapnic ventilated group.

### Effects of hypercapnia on ventilator-induced diaphragm dysfunction

In line with previous studies in animals and humans the present findings confirm that diaphragm weakness upon prolonged mechanical ventilation is accompanied by increased levels of inflammatory mediators in the diaphragm, activation of proteolytic pathways and loss of contractile proteins [1,3,4,22-26].

Hypercapnic acidosis has been shown to impair diaphragm function [27-30]. Very recently however, Jung *et al*. showed that hypercapnic acidosis protects against diaphragm dysfunction in piglets after 72 hours of mechanical ventilation [9]. In apparent contrast, we observed a decreased force in single fibers after 18 hours of hypercapnic mechanical ventilation. Of note, Jung *et al*. used an *in vivo* model of diaphragm function, by stimulating phrenic nerves while the present study looked specifically at contractile proteins and their function. A plausible explanation for this discrepancy is that recovery of diaphragm function upon hypercapnia becomes significant after 18 hours of mechanical ventilation. Interestingly, in the study by Jung *et al*. diaphragm function was also decreased after 12 hours in both normal and hypercapnic ventilated groups [9]. Although that study clearly showed a potential beneficial effect of hypercapnia after 72 hours of mechanical ventilation, the mechanisms by which hypercapnia exerts these effects were not studied [10]. An important finding of the present study is that hypercapnia completely prevented mechanical ventilation-induced loss of myosin (Figure 2b). Preservation of myosin most likely resulted from reduced degradation, as mechanical ventilation under hypercapnic conditions was associated with reduced 20S proteasomal activity and a trend of increased ratio of ubiquitinated myosin over total myosin (Figure 4a,b). Of note, the correction for total myosin presumably underestimates the actual amount of ubiquitinated myosin in MV + H rats, since the concentration of myosin is increased in this group. We observed a trend of decreased myosin content per muscle weight in ventilated rats, but not in hypercapnic ventilated rats. Since myosin is one of the major proteins in skeletal muscle, correcting myosin content for total muscle weight is expected to underestimate the loss of myosin. Therefore we chose to analyze myosin loss more precisely by measuring myosin concentration (that is the amount of myosin per muscle volume) in single fibers. Remarkably, concentration of myosin in diaphragm single fibers of hypercapnic ventilated animals was even higher than in controls. This can be explained by the fact that the CSA was slightly decreased in the hypercapnic ventilated group compared with the control group (Figure 2c). Accordingly, myosin concentration increases when myosin content remains stable, but the volume of the muscle fiber, reflected by CSA, decreases. These data suggest that loss of myosin and loss of muscle fiber circumference are likely not tightly linked [31]. A recent paper also found myosin concentration levels above control values in the diaphragm of rats with pulmonary hypertension. This was also explained by a disproportional reduction of CSA [32]. Preservation of myosin in the hypercapnic ventilated group, however, did not protect the diaphragm muscle from reduced force generation, implicating more dysfunctional myosin in hypercapnic ventilated rats. This discrepancy is most likely the result of posttranslational modifications of contractile proteins. Indeed, the antioxidant DTT increases force-generating capacity of diaphragm fibers from the MV + H group, indicating that oxidative protein modifications play a role in loss of force induced by controlled mechanical ventilation under hypercapnic conditions. Notably, DTT did not improve force generation in muscle fibers in the group ventilated under normocapnic conditions (Figure 6a). There are two reasonable explanations for the different effect of DTT on normocapnic and hypercapnic fibers. First, as hypercapnia reduced proteasomal activity, degradation of posttranslationally modified (that is oxidized) myosin in the diaphragm is attenuated. In line with that, hypercapnic ventilation tended to increase levels of ubiquitinated myosin. In both ventilated groups, an increase of LC3B-II protein suggests activation of autophagy. However, the autophagy marker beclin-1 was not increased. Such a discrepancy has also been observed recently in the diaphragm in septic mice [33], suggesting that autophagy can occur independent of beclin-1.

Second, hypercapnia may be associated with oxidative stress. Indeed, Arbogast *et al*. demonstrated that elevated CO_2_ promotes oxidant activity in diaphragm muscle bundles [34]. We attempted to detect oxidative modifications of muscle proteins by measuring level of HNE. However, we did not see any difference between groups in HNE levels, which does not exclude that other oxidative modifications, for example formation of disulfide bonds, have occurred.

In the present study, hypercapnic ventilated animals showed an acidemia during most time of mechanical ventilation period (Figure 1a). The question that remains is if the effects on the diaphragm observed in the present study are the result of hypercapnia or the combination of hypercapnia and acidemia. We have recently concluded that most beneficial effects appear to be pH mediated in both *in vivo* and *in vitro* experiments, although synergistic effects of acidemia and hypercapnia have also been observed [8]. Besides its direct effects on muscle function, hypercapnia has systemic effects. For example, by a rightward shift of the oxyhemoglobin dissociation curve, oxygen release to the tissues is facilitated [8] and by sympathetic stimulation increasing cardiac output and MAP [8,9]. Unexpectedly, we observed a significantly decreased MAP in hypercapnic ventilated animals. Whether systemic effects of hypercapnia could affect diaphragm function during controlled mechanical ventilation is, however, uncertain.

#### Effect of cytokines in muscle and hypercapnia

Recent work from our laboratory showed that 8 hours of mechanical ventilation induces an inflammatory response in the mouse diaphragm [4]. The current study confirms this observation in the mechanically ventilated group. Hypercapnia largely abolished the increases in levels of inflammatory cytokines in the diaphragm of ventilated rats (Figure 3). Attenuating the inflammatory response by hypercapnia might protect the diaphragm from atrophy during mechanical ventilation. Previous studies have shown that hypercapnia dampens the inflammatory response, probably through inhibition of nuclear factor kappa B (NF-κB), in acute lung injury [6,8,35]. The question that remains is whether myosin preservation and depression of proteasome activity in the diaphragm of the hypercapnic ventilated group is mediated by attenuation of the inflammatory response or by a direct effect of hypercapnia on NF-κB inhibition. Both TNF-α and IL-1beta have been associated with skeletal muscle atrophy [36-38]. TNF-α induces protein loss in cultured C2C12 skeletal muscle cells [37] and exposure of skeletal muscle cells to IL-1beta for 48 hours induces muscle atrophy [36]. Of note, although IL-10 is an anti-inflammatory cytokine, its production is induced by proinflammatory mediators and therefore changes in IL-10 levels, as observed in the current study, can also be considered as a surrogate marker of the magnitude of the proinflammatory response [39,40].

#### Clinical relevance

Hypercapnic acidosis has been shown to attenuate ventilator-induced lung injury and inflammation [8]. The present study shows that therapeutic hypercapnia may also protect the diaphragm from adverse events associated with controlled mechanical ventilation. This is of potential clinical importance, since respiratory muscle dysfunction frequently occurs in mechanically ventilated patients and is associated with prolonged weaning, increased morbidity and mortality in ICU patients [41-43]. Besides training, no specific treatment is currently available for muscle weakness in the critically ill. Therefore, therapeutic hypercapnia during mechanical ventilation in order to prevent ventilator-induced diaphragm weakness deserves further study.

Our study also generates new hypotheses on the role of antioxidants in the prevention of ventilator-induced respiratory muscle dysfunction. Betters *et al*. have shown that administration of antioxidants at time of initiation of mechanical ventilation is effective in the prevention of diaphragm dysfunction [12]. Our data indicate that once ventilator-induced diaphragm dysfunction has occurred antioxidants might not be effective (Figure 6a), unless mechanical ventilation is performed under hypercapnic conditions. This study indicates that the timing of antioxidant administration may be of critical importance as well as the associated conditions (that is, the presence of hypercapnia).

## Conclusions

The present study shows that mechanical ventilation under hypercapnic conditions protects the diaphragm from ventilator-induced diaphragm atrophy and enables antioxidants to restore diaphragm muscle force after 18 hours of mechanical ventilation.

## Key messages

• Mechanical ventilation under hypercapnic acidosis attenuates ventilator-induced diaphragm atrophy, inhibits proteolysis, and reduces diaphragmatic inflammation.

• Hypercapnic acidosis enables antioxidants to restore diaphragm muscle force generation.

## Abbreviations

CON: control group; CSA: cross-sectional area; DTT: dithiothreitol; HNE: 4-hydroxy-2-nonenal; IL: interleukin; KC: keratinocyte-derived chemokine; LC3B-II: light chain 3B-II; MAP: mean arterial pressure; MV + H: group of 18-hour mechanically ventilated rats with hypercapnia; MV: group of 18-hour mechanically ventilated rats; MyHC: myosin heavy chain; NF-κB: nuclear factor kappa B; OD: optical density; TNF-α: tumor necrosis factor alpha.

## Competing interests

The authors declare that they have no competing interests.

## Authors’ contributions

WS and HvH contributed to designing the study, acquiring, analyzing and interpreting the data and writing the manuscript. MK helped to design the study and contributed to revising the manuscript. ML and GAA carried out the biochemical analyses and contributed to revising the manuscript. PNRD participated in the design of the study and contributed to revising the manuscript. GS and JvdH participated in the design of the study, the analysis of the data and contributed to revising the manuscript. LH conceived of the study and participated in its design, coordination, interpretation of the data and helped to draft the manuscript. All authors read and approved the final manuscript.
